# Using ESTs for phylogenomics: Can one accurately infer a phylogenetic tree from a gappy alignment?

**DOI:** 10.1186/1471-2148-8-95

**Published:** 2008-03-26

**Authors:** Stefanie Hartmann, Todd J Vision

**Affiliations:** 1 Department of Biology, University of North Carolina, Chapel Hill, NC 27599, USA; 2 Institute for Biochemistry and Biology, Karl-Liebknecht-Strasse 24-25, University of Potsdam, 14476 Potsdam, Germany

## Abstract

**Background:**

While full genome sequences are still only available for a handful of taxa, large collections of partial gene sequences are available for many more. The alignment of partial gene sequences results in a multiple sequence alignment containing large gaps that are arranged in a staggered pattern. The consequences of this pattern of missing data on the accuracy of phylogenetic analysis are not well understood. We conducted a simulation study to determine the accuracy of phylogenetic trees obtained from gappy alignments using three commonly used phylogenetic reconstruction methods (Neighbor Joining, Maximum Parsimony, and Maximum Likelihood) and studied ways to improve the accuracy of trees obtained from such datasets.

**Results:**

We found that the pattern of gappiness in multiple sequence alignments derived from partial gene sequences substantially compromised phylogenetic accuracy even in the absence of alignment error. The decline in accuracy was beyond what would be expected based on the amount of missing data. The decline was particularly dramatic for Neighbor Joining and Maximum Parsimony, where the majority of gappy alignments contained 25% to 40% incorrect quartets. To improve the accuracy of the trees obtained from a gappy multiple sequence alignment, we examined two approaches. In the first approach, alignment masking, potentially problematic columns and input sequences are excluded from from the dataset. Even in the absence of alignment error, masking improved phylogenetic accuracy up to 100-fold. However, masking retained, on average, only 83% of the input sequences. In the second approach, alignment subdivision, the missing data is statistically modelled in order to retain as many sequences as possible in the phylogenetic analysis. Subdivision resulted in more modest improvements to alignment accuracy, but succeeded in including almost all of the input sequences.

**Conclusion:**

These results demonstrate that partial gene sequences and gappy multiple sequence alignments can pose a major problem for phylogenetic analysis. The concern will be greatest for high-throughput phylogenomic analyses, in which Neighbor Joining is often the preferred method due to its computational efficiency. Both approaches can be used to increase the accuracy of phylogenetic inference from a gappy alignment. The choice between the two approaches will depend upon how robust the application is to the loss of sequences from the input set, with alignment masking generally giving a much greater improvement in accuracy but at the cost of discarding a larger number of the input sequences.

## Background

Advances in high-throughput sequencing and computational power have enabled phylogenetic analyses on an unprecedented scale [1-3]. Large-scale phylogenetic studies of gene families can be used to clarify the relationships among organisms [4,5] or to study the evolution and function of the genes themselves [6,7]. Such analyses are frequently restricted to only those genes for which a full-length sequence is available, because partial sampling of a gene family may diminish the accuracy of downstream applications such as orthology assignment [8,9] and gene-tree reconciliation [10]. Thus, it would be desirable for many applications to sample additional gene family members from the much larger number of partial gene sequences that are available.

**Figure 1:**
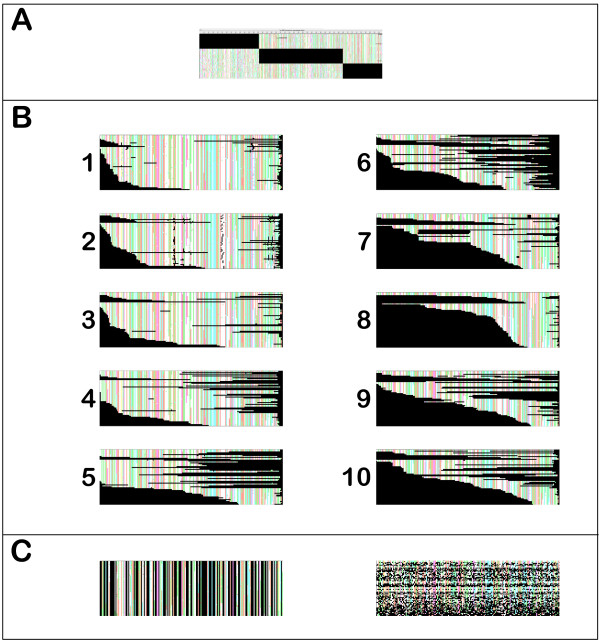
**Patterns of gappy alignments**. Rows represent individual sequences, and black regions indicate missing data. A. A concatenated alignment of three genes, not all of which have been obtained from all species. B. Gap patterns used for the artificial alignments. Each gap pattern is based on a single gene family in the Phytome database. The total percentage of missing amino acids for each alignment is as follows. 1: 14%; 2: 21%; 3: 20%; 4: 29%; 5: 46%; 6: 54%; 7: 55%; 8: 60%; 9: 56%; 10: 58%. C. Example of column-deleted and random-deleted control alignments. The examples shown contain the same percentage of missing amino acids as gap pattern 4 in panel B.

Partial gene sequences are primarily derived from expressed sequence tags (ESTs) [11]. ESTs are generated by isolating bulk mRNA from a given tissue, reverse-transcribing the mRNAs into cDNAs, cloning the cDNAs into a vector, and then sequencing many individual clones from one or both ends using universal primers. Because overlapping segments of the same gene may be sequenced multiple times, it is customary to assemble overlapping ESTs into a single consensus *unigene*. Since even unigenes rarely cover the full-length of their corresponding transcript sequences, multiple sequence alignments derived from EST unigenes are often gappy. The gaps tend to be clustered at the beginning and/or the end of each unigene sequence, and the positions that are missing often overlap but do not exactly correspond between unigenes (Figure 1). Typically, an alignment that contains EST unigenes will also contain at least some genes that are full-length: either because a complete genome sequence is available for the species, because the cDNA for that particular gene has been sequenced, or, more rarely, because the entire gene is spanned by the unigene. We also note that other high-throughput sequencing techniques [12] can lead to similar patterns of gappiness and may eventually contribute a large proportion of the partial gene sequences in public databases.

Gappy alignments present a number of difficulties for phylogenetic analysis. First, phylogenetic inference is statistically compromised by a lack of data. Second, different regions in an alignment do not necessarily have the same intrinsic substitution rate due to differences in the strength of purifying and positive selection. This may introduce bias if these different regions are more complete in some sequences than in others. One may overestimate the phylogenetic distance between two sequences that overlap in a fast-evolving portion of the alignment and underestimate the phylogenetic distance between two sequences that overlap in only the slowly-evolving regions. Finally, for phylogenetic methods that utilize a pairwise distance matrix, it may not even be possible to compute all pairwise distances due a lack of overlap between some pairs of sequences.

The debate about how to deal with missing data in phylogenetic analysis (omit, ignore, impute, or model) is not new [13-23]. A number of these studies have focused on incomplete gene sampling when evaluating a superalignment approach to an incomplete multigene dataset [20,24,25]. The general conclusions have been that the relative amount of missing data is not the most important factor in determining whether a correct phylogeny can be computed. Instead, the absolute amount of available, informative data within an alignment is more important. Accurate phylogenies can be obtained even if up to half the sequences within an alignment contain up to 90% missing data [24]. However, the pattern of gappiness seen in a superalignment (concatenated alignment), where some genes are missing from some taxa, differs from the pattern of gappiness due to partial gene sequences. In the former case, the boundaries of the missing data blocks strictly coincide among subsets of the sequences in a superalignment, while in the latter case the gaps are staggered (Figure 1). To our knowledge, it has not yet been determined whether the patterns of missing data in concatenated alignments and within EST-like alignments have comparable effects.

Here, we use simulated data to specifically examine the effects of EST-like gappiness on phylogenetic accuracy. Our analyses are based on correct alignments, while in real life situations, correct alignments are often unknown and hard to compute, especially in regions that contain gaps. Our alignments therefore represent the "ideal cases" of true alignments. Using these simulated data, we explore the contribution of a variety of factors by comparing alignments that differ with respect to number of sequences, tree topology, alignment length, and gap pattern. We have found that EST-like gappiness results in lower accuracy than the pattern of missing data obtained with incomplete gene sampling in a superalignment, even in the absence of alignment error. To address this problem, we have compared two methods designed to increase the accuracy of phylogenetic inference from a gappy alignment: alignment masking and alignment subdivision. In alignment masking, certain alignment columns and input sequences are excluded from phylogenetic analysis. In real data, alignment masking may also be used to ensure the positional homology of the columns that remain, to eliminate positions that appear to have undergone multiple substitutions, and to exclude systematically misaligned sequences in an automated alignment workflow. Although alignment masking is most often based on subjective criteria, systematic approaches have also been developed [26-28,51]. Because masking comes at the necessary expense of failing to include some fraction of the input sequences, we have also explored an alignment subdivision approach in which the data are partitioned into subalignments with minimal gaps. The phylogenetic tree is computed from a combined distance matrix estimated by weighting the data from each subalignment and imputing missing data. We have found that both approaches can improve the accuracy of phylogenies computed from gappy alignments.

## Results

In order to measure phylogenetic accuracy, we used a set of simulated gappy protein alignments that resemble those derived from EST data (see Methods for details). Briefly, 540 full-length protein sequence families varying with respect to their lengths, number of sequences, substitution rate, and tree topology were generated by the Rose software package [29], and the true multiple sequence alignments and phylogenetic topologies were recorded. To simulate patterns of missing data comparable to those obtained with EST unigenes, ten gap patterns were then chosen based on protein sequence alignments in the Phytome plant comparative genomics database [30] and applied to each of the full simulated alignments. In order to separate the effects of EST-like gappiness from the effects of missing data *per se*, we generated two types of control alignments. For these, the same amount data were removed as for the EST-like gappy alignments, but the residues to be deleted were either concentrated within alignment columns or randomly distributed throughout the alignment (from here on referred to as *column-deleted *and *random-deleted*, respectively).

### Alignments with no missing data

**Figure 2:**
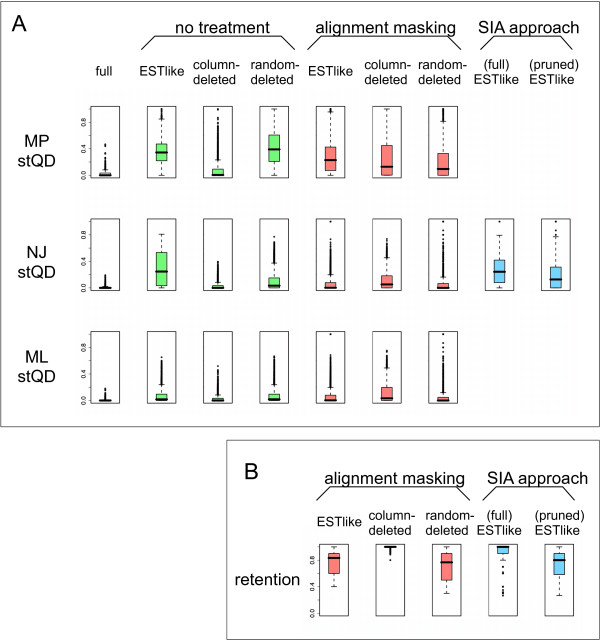
**Phylogenetic accuracy and the retention of sequences**. **A**. Distribution of standardized quartet distances between estimated phylogenies and the corresponding true trees. Leftmost column: full alignments with no gap pattern applied. Green: gap pattern applied, phylogeny inferred directly (without the use of masking or SIA). Red: alignment masking. Blue: SIA. **B**. Proportion of sequences retained per family. Boxplots show the median (horizontal black bar) and interquartile range (colored boxes).

We first tested whether the 540 full simulated alignments contained sufficient phylogenetic signal to recover the true trees using Neighbor-Joining (NJ), Maximum Parsimony (MP), and Maximum Likelihood (ML). All comparisons of true trees with estimated trees are given as standardized quartet distances *stQD*, which is a measure of how many quartets, or sets of four sequences, differ in topology between two phylogenies [31,32]. Our *stQD *thus ranges from zero (no topological disagreement with the true tree) to one (no quartets accurately inferred). Results are shown in Figure 2A. The standardized quartet distances for MP had a median of 0 and a mean of 0.0302 (i.e., on average, the topology of 3.02% of all possible subtrees of size 4 differed between the inferred and the true tree). For NJ, *stQD *had a median of 0 and a mean of 0.0134. For ML, *stQD *had a median of 0 and a mean of 0.0105. Taken together, these results confirm that the data in the full simulated alignments are sufficient to allow recovery of very nearly true trees, most of the time, using all three phylogenetic inference approaches.

### Alignments with missing data

We then evaluated the extent to which missing data within these alignments compromise phylogenetic accuracy, and the effect of EST-like gappiness relative to other patterns of missing data. NJ, MP, and ML phylogenies were computed for the 5400 EST-like alignments, the 5400 column-deleted alignments, and the 5400 random-deleted alignments (green boxplots in Figure 2A). The *stQD *values for the EST-like gappy alignments were substantially higher than for the full alignments, with a median approaching 0.4 in the case of MP. The median *stQD *for the column-deleted alignments was zero or very close to zero, although the missing columns did lead to some lengthening of the upper tail of the distribution of *stQD *values. The difference in phylogenetic accuracy was less pronounced between EST-like and random-deleted alignments, and the rank order of performance among NJ, MP, and ML was similar between these treatments. The maximum *stQD *for MP in the random-deleted alignments was 1.0, meaning that no quartets in the true tree were accurately inferred.

Overall, ML had the highest accuracy and the least sensitivity to missing data. However, the performance of the different algorithms depended to some extent on the nature of the alignments used. In particular, the gap pattern had a major effect on the relative performance of the different phylogenetic methods. For ≈13.5% of the EST-like alignments, NJ resulted in the most accurate phylogeny. Of these, approximately 80% were generated using gap patterns 1, 2, or 8. Of the ≈37% EST-like alignments for which NJ resulted in the most inaccurate trees, 25% each were generated using gap patterns 6 and 9. For approximately ≈2.5% of the EST-like alignments, MP yielded the best tree. Of these, 90% were the shortest alignments (50 amino acids), 82% were the alignments with only 10 sequences, and approximately 75% were generated using gap patterns 7, 8, or 9.

### Alignment masking

One common approach to preprocessing a gappy alignment prior to phylogenetic inference is to mask the alignment such that only columns and sequences containing sufficient and reliable phylogenetic information are included. Here, we used a simple algorithm we have named REAP (REducing Alignments prior to Phylogenetic reconstruction) [30], which is designed to mask (i) columns containing many gaps and/or highly diverse amino acids and (ii) sequences that either have little overlap with other sequences or appear to be systematically misaligned. In our simulated sequence families, since there are no actual alignment errors in the simulated dataset, REAP is discarding true phylogenetic information in the data.

**Figure 3:**
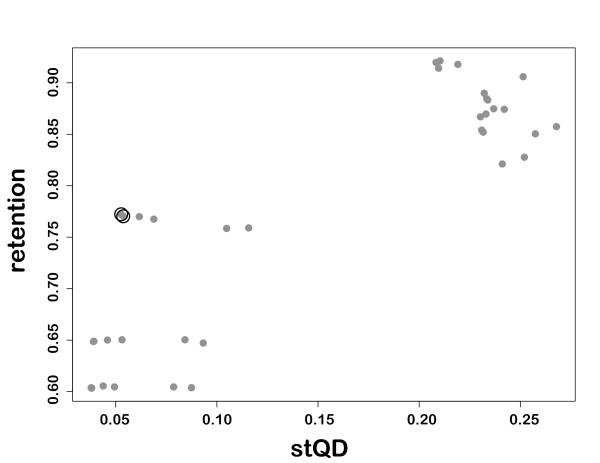
**Relationship between phylogenetic accuracy and the proportion of sequences retained using REAP**. The two REAP runs with the parameters determined to be optimal for the simulated data are indicated by black circles around the data points.

To determine the appropriate masking parameters to use in this study, we conducted a factorial experiment in which 36 parameter combinations were tested (see Methods). We recorded the number of sequences retained after masking, and the *stQD *of the NJ tree. Because of the prohibitive amount of time that would have been required, MP and ML trees were not calculated. We analyzed the 4901 (90.8%) of the alignments for which phylogenies could be computed for all 36 parameter combinations (i.e. at least four sequences were retained). Evaluation of the computed phylogenies showed that the exclusion of sequences with varying proportions of gaps had much larger effects on phylogenetic accuracy than the exclusion of gappy columns. Analysis of variance demonstrated that all parameters had significant main effects, and a variety of higher-level interactions were significant (results not shown). The parameter with the largest marginal effect on both accuracy and retention was *g*_*s*_, the maximum proportion of gaps allowed in a sequence. Overall, we found a clear trade-off between the accuracy of the phylogeny obtained and the proportion of sequences eliminated from the alignment (Figure 3). Parameter settings of *s*_*c *_= 0.5, *g*_*c *_= 0.5 *g*_*s *_= 0.5 (see Methods) were determined to strike the best compromise between topological accuracy and sequence retention, regardless of window size. Thus, unless otherwise noted, we used these parameters with a window size of six for subsequent analyses.

Using the selected parameters, we applied REAP to all three sets of gappy alignments (EST-like, column-deleted, and random-deleted) and then computed phylogenies using NJ, MP, and ML (Figure 2A, red boxplots). Quartets involving sequences that were not retained by REAP did not contribute to the calculation of *stQD*. For EST-like alignments, masking greatly improved accuracy for all three phylogenetic methods. The greatest improvements were obtained using NJ and ML (where the median *stQD *dropped from 0.246 to 0.002, and from 0.017 to 0.002, respectively). MP trees improved less dramatically (the median *stQD *dropped from 0.3438 to 0.2286). Results were qualitatively similar for random-deleted alignments. By contrast, masking of column-deleted alignments led to less accurate trees using all three phylogenetic methods. For NJ and ML, the masked column-deleted alignments resulted in less accurate trees, on average, than the masked EST-like alignments. This may be due to the nearly complete retention of sequences in the masked column-deleted alignments compared to approximately 80% retention in the masked EST-like alignments (Figure 2B). As can also be seen in Figure 2B, levels of retention were similar for EST-like and random-deleted alignments.

To determine which factor(s) in the simulated alignments (alignment length, evolutionary rate, number of sequences, tree topology, and gap pattern) most affected phylogenetic accuracy, we performed analysis of variance on *stQD *of the NJ, MP, and ML trees after alignment masking (Table 1). This analysis showed that overall, no single parameter predominantly influenced the *stQD *values. Instead, all parameters had significant main effects. Furthermore, several two-way and three-way interactions were significant, as was the four-way interaction of number of sequences, tree topology, alignment length, and gap pattern.

**Table 1:** ANOVA with response variable *stQD*.

Parameters	NJ	MP	ML	SIAa	SIAb
length	***	***	***	***	***
rate	*	***	**	*	
seqs	***	***	***	***	***
topol	***	***	***	**	***
gap	***	***	***	***	***
length:rate	**	**			
length:seqs	***	***	***	*	***
rate:seqs		***	***		
length:topol			***	***	**
rate:topol	***	***	***	***	**
seqs:topol	***	***	***	***	**
length:gap	***	***	***	***	**
rate:gap				***	
seqs:gap	***	***	***	***	***
topol:gap	***	***	***	***	***
length:rate:seqs					
length:rate:topol	***	*	**	**	**
length:rate:topol		***	***	***	***
rate:seqs:topol					
length:rate:gap					
length:seqs:gap	***	***	***	***	***
rate:seqs:gap				***	
length:topol:gap	**	***	***	***	***
rate:topol:gap					
seqs:topol:gap		***	***	***	***
length:rate:seqs:topol				*	
length:rate:seqs:gap				*	
length:rate:topol:gap					
length:seqs:topol:gap		***	*	***	***
rate:seqs:topol:gap					
length:rate:seqs:topol:gap					

*p *≤ 0.001***, *p *≤ 0.01**, *p *≤ 0.05*; parameters: length = alignment length; rate = evolutionary rate; seqs = number of sequences in the alignment; topol = tree topology of true tree; gap = gap pattern applied to full alignment. SIAa refers to the full SIA trees, while SIAb refers to the pruned SIA trees.

### Statistically correcting for missing data

**Figure 4:**
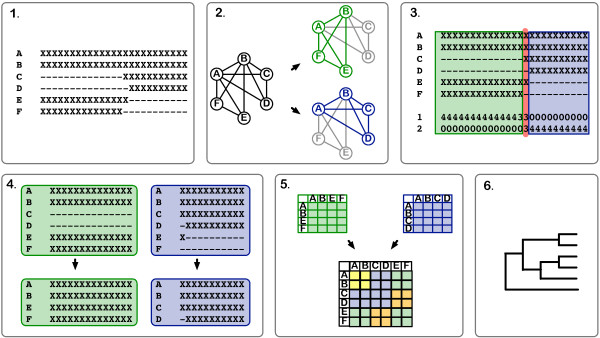
**Overview of SIA method**. 1. Initial gappy alignment (The example shows an alignment of six sequences (A-F). "X" represents any amino acid; "-" represents a gap or missing data.); 2. The overlap-graph and two maximal cliques (green and purple); 3. Assignment of columns to cliques. The red column is placed in the smaller of the two cliques; 4. Two subalignments corresponding to the two cliques; 5. The resulting submatrices, and the combined matrix, of pairwise distances. Yellow cells are represented in both the green and purple submatrices. Orange cells must be imputed. ; 6. The phylogenetic tree estimated from the combined distance matrix. See text for details.

In contrast to the approach of alignment masking, in which data are excluded from the analysis, it might be possible to improve phylogenetic accuracy by statistically modeling the missing data. The approach we examined in this study is called "SIA", for Subdividing Incomplete Alignments (F. Cheng, S. Hartmann, M. Gupta, J. Ibrahim, and T. Vision, in prep.) and is illustrated in Figure 4 (see Methods for details). SIA attempts to estimate the distance matrix that would be obtained in the absence of missing data, thereby improving phylogenetic accuracy (using NJ or another distance-based phylogenetic method) without sacrificing sequence retention. In brief, the full multiple sequence alignment is partitioned into subalignments that have little to no missing data, and a distance matrix is computed for each subalignment. A phylogenetic tree is then computed from a combined distance matrix estimated by weighting the distance data from each subalignment and imputing missing data.

In our implementation of SIA, EST-like alignments yielded an average of four subalignments (and a maximum of 16), with a mean subalignment length of 58.9 columns. Column-deleted alignments predictably yielded only one subalignment, while the random-deleted alignments yielded a prohibitively large number of potential subalignments ($\bar{x}$ = 53.4) and thus could not be analyzed for comparison. NJ phylogenies could be computed for 5,237 of the EST-like families. Of the 163 families that were discarded, 80 with gap pattern 6 and two with gap pattern 10 had too many missing pairwise distances to impute values using the four-point metric (see Methods). The remaining 81 families were discarded by REAP due to insufficient alignment data.

The median percentage of sequences retained within a family was 100%, while the median *stQD *was 0.24 (Figure 2). Thus, the phylogenetic accuracy obtained by SIA is comparable to that obtained by masking followed by MP, but with substantially higher retention of sequences. Since the SIA trees include more sequences than the masked trees, and the additional sequences might be among the most problematic to place accurately in the tree due to their reduced length, a fairer comparison would be to compute *stQD *for a pruned SIA tree having the same set of sequences as the masked tree. Pruned SIA trees had a median *stQD *of 0.13, which, though better than unpruned SIA trees, is still less accurate on average than obtained by masking followed by NJ or ML. To determine which (combinations of) parameters used to generate the alignments significantly affected the phylogenetic accuracy, we performed ANOVA on *stQD *for both full and pruned SIA trees. The results generally agreed with those from alignment masking: all simulation parameters had significant main effects and contributed to higher-level interactions (Table 1; SIAa: full trees, SIAb: pruned trees).

### Algorithm performance relative to simulation parameters

For a more detailed comparison of results for the SIA approach and alignment masking followed by either NJ, MP, or ML, we compared *stQD *among the 3,527 families for which we were able to compute phylogenies for all four methods. We also examined variation in *stQD *among the ten gap patterns. We found that alignment masking, when followed by either NJ or ML, resulted in the most accurate phylogeny in ≈72% of cases (approximately 36% each for NJ and ML), and that alignments with 60 sequences were over-represented in this set. Alignment masking followed by MP resulted in the best trees for ≈9% of the families. Of these, almost 70% were alignments of length 50, nearly 70% contained 10 sequences, and approximately 60% had gap patterns 5 or 7 applied to them. In only ≈7% of the families, the SIA approach resulted in the most accurate phylogeny. Of these, 80% were the shorter alignments, 70% were those to which gap pattern 8 had been applied, and 64% had only 10 sequences. In approximately 20% of all families, no difference was observed across any of the methods. These were in most cases perfect phylogenies (*stQD *= 0). All of these were alignments of 10 sequences, and masks 9 and 10 were overrepresented among them (36% and 33%, respectively).

## Discussion

ESTs and other partial gene sequences are the predominant source of sequence data for a large and taxonomically diverse set of species. These sequences are tremendously valuable for gene discovery, genome annotation, comparative genomics, marker development, and a variety of other uses [11,33]. However, for studies of gene family evolution or for large-scale analyses of gene families, one must contend with the large amount of missing data in alignments derived from partial sequences. For example, of the ≈27,000 families in the Phytome database [30] for which there are three or more sequences, the average proportion of alignment gaps is 37%.

Are these missing data really a problem? We found that it was possible to recover accurate trees from alignments in which the missing residues were clustered into columns. Even though half of the simulated alignments had between 50% and 60% missing data, the median *stQD *for the NJ and ML trees were 0, and the median *stQD *for the MP trees was 0.004. These results confirm that the presence of missing data itself does not lead to an incorrect phylogeny as long as sufficient data is available for the analysis [20,22,24,25].

However, EST-like gappy alignments appear to be qualitatively different. When the same amount of missing data was distributed in a pattern typical of EST unigenes, phylogenies were much less accurate: mean *stQD *for trees computed from these alignments ranged from 0.17 for ML to 0.34 for MP. When using NJ, the phylogenetic accuracy was even lower for the EST-like gappy alignments than for alignments in which the same number of residues were randomly deleted. One explanation for these results is that for the random-deleted alignments, there is at least some overlap between all the pairs of sequences. For the EST-like gappy alignments, on the other hand, it is common for some pairs of sequences to share no columns in which data are present (e.g. see gap patterns 5, 6, 9 and 10), and thus no distance can be computed. This poses particular problems for distance methods. For example, PHYLIP reports a distance of "-1.0" for any two sequences that do not overlap in the input alignment. This is taken at face value during execution of the NJ algorithm, leading to a systematic bias toward overly close relationships between sequences in the tree as a result of the lack of overlap between them. The importance of the distribution, and not just the amount, of missing data, was shown earlier in a different context by Wiens [24]. In that study, lower accuracy was obtained when missing genes were randomly distributed among the sampled taxa, compared to data sets in which the missing genes were restricted to monophyletic subsets of taxa.

We have shown that one can improve phylogenetic accuracy by taking either one of two diametrically opposed approaches. In the first approach, one excludes gappy columns and sequences from the analysis through alignment masking. In our implementation of masking (REAP), we mimic the way it would be performed on real data by also excluding columns and rows that show evidence of misalignment, even though there is no alignment error in our simulation. Most of the trees computed from masked alignments using either NJ or ML methods were comparable to those computed from alignments without any missing data (mean *stQD *of 0.0022 and 0.0026 vs. 0.0 for full alignments). Even for MP tress, alignment masking was able to improve the trees approximately to the level of unmasked NJ trees (*stQD *of 0.2286). While this may be due solely to the removal of gaps, it may also reflect the removal of alignment positions that have undergone multiple substitutions, thus making the phylogenetic signal clearer in those that remain. Either way, one cannot escape the paradox that the phylogeny is made more accurate by ignoring error-free alignment input. Another important point is that alignment masking comes at the necessary expense of failing to retain all the sequences. On average, 27% of the sequences within an EST-like alignment were excluded by masking in our experiments.

A very different approach is to attempt to model the missing data, which we have done through a technique we call alignment subdivision. Relative to masking, we found that our implementation of alignment subdivision (SIA) was able to retain a much higher proportion of the sequences; the median proportion of retained sequences using SIA was 100%. SIA generally, though not universally, led to more accurate trees than those computed directly from the gappy alignment. The greatest improvements in accuracy under SIA were seen in those families that had many subalignments. Where incomplete alignments were divided into 12 or more subalignments, SIA resulted in a more accurate phylogeny in almost all cases. On the other hand, when there were only two subalignments, the phylogeny computed directly from the original alignment was more accurate two-thirds of the time. Perhaps not surprisingly, the number of subalignments was closely associated with the gap pattern used in the simulation. Gap patterns 1, 2, 3, and 8 typically resulted in only one to four subalignments, while gap patterns 6, 9, and 10 typically resulted in a much larger number; thus, certain gap patterns are intrinsically more likely to see an improvement under SIA than others.

The improvement in phylogenetic accuracy was generally much higher with masking than with subdivision. NJ trees computed from EST-like alignments were over 100-fold more accurate with alignment masking (*stQD *= 0.002) than when directly computed (*stQD *= 0.246). The same differential was only about two-fold when using SIA (*stQD *= 0.127). The phylogenetic accuracy using SIA was thus comparable to the masked MP trees and the unmasked ML trees. Furthermore, the SIA approach is computationally laborious. Taken together, our results suggest that alignment masking is the preferred approach when the distribution of missing data is EST-like in nature.

While it appears from our results that alignment masking is not necessary when ML is used to infer the phylogeny, this may reflect the lack of alignment error in the simulated data. Although under some circumstances, the choice of phylogenetic inference method is known to have a major effect on phylogenetic accuracy [34], previous studies have shown that both alignment accuracy [35-37] and the ratio of phylogenetic signal to noise in the alignment [38] can be even more important than the choice of phylogenetic method. While we have not studied the effects of misalignment due to using partial gene sequences as input, we suggest that alignment error is likely to improve the relative performance of masking.

In modeling the missing alignment data, we have estimated the distance matrix that we would expect to see in the absence of missing observations. To further develop and optimize the SIA method, other approaches for combining subalignments can be tested in future studies. For example, we have imputed pairwise distances that could not be computed from the submatrices using a four-point metric [18,39]. Future implementations of SIA could be improved by incorporating a three-point metric or a weighted least-squares imputation [18,23,40]. However, because only 17.5% of the cells in the combined matrices were missing, we expect the difference in imputation quality to have only minor effects on the results. Alternative approaches that model the missing alignment data probabilistically or by imputation would allow more accurate (likelihood or Bayesian) phylogenetic techniques to be applied while still retaining all the input sequences. Another interesting approach would be to infer phylogenies separately for each subalignment and then calculate a supertree for the full dataset [41].

## Conclusion

Given that the vast majority of publicly available sequence data from complex genomes is derived from large-scale partial gene sequencing projects, it would be a serious handicap to limit phylogenetic analyses to alignments derived only from full-length sequences. However, we have shown that the particular pattern of gappiness found in alignments of partial gene sequences needs to be handled with care in order to obtain accurate phylogenies. Both masking and model-based approaches to missing data show potential for improving the accuracy of the trees obtained from gappy alignments. Their performance will have to be compared to other approaches to deal with incomplete alignments [14,23]. Such methods will be critical for the application of techniques that rely upon large numbers of accurate gene trees, as is common in phylogenomics [4,6].

## Methods

### Generating simulated alignments

We used the software Rose [29] to simulate 540 families of evolutionarily related and correctly aligned protein sequences. Each sequence family member was derived by successive substitution from a common ancestor along a defined evolutionary tree. Insertions and deletions were disallowed. Substitution probabilities were given by a PAM transition matrix [42]. As a starting sequence we used the first 50, 200, or 500 amino acids from the *Capsicum chinense *phenylalanine ammonia-lyase protein (Genbank Accession AAC33966.1). Our sequence families differed with respect to four factors (see [29] for definitions): length (50, 200, or 500 amino acids), the number of sequences (10 or 60), substitution rate (uniformly fast with a substitution probability of 1.0, uniformly slow with a substitution probability of 0.5, or a mosaic with probability of 1.0 and 0.5 alternating every 15 residues), and tree topology (fully asymmetric, fully symmetric/balanced, or a randomly generated intermediate). The chosen sequence lengths represent typical ranges found in sequence family databases such as Phytome [30]. Branch lengths of the input trees for the software Rose were adjusted such that the average relatedness for all pairwise sequences of a given sequence family was the same (250 PAM). Variability of sequences and sequence-regions was then reduced where the mutation probability was set to 0.5. For each of the 54 parameter combinations, we generated 10 replicate families that sampled independent random substitutions.

### Simulating missing data

#### EST-like alignments

To simulate patterns of missing data comparable to those obtained with EST unigenes, we selected ten multiple sequence alignments from Phytome [30], which contains multiple sequence alignments of both full-length proteins and protein sequences inferred from EST unigenes. The ten selected alignments ranged in length from 155 to 648 amino acids and from 14% to 60% missing residues (Figure 1). We selected alignments that contain exactly 60 sequences and that represent the typical range of missing data found in sequence alignments that contain EST data. With respect to other parameters (e.g., species representation or gene function), these alignments were chosen randomly. Gap patterns seen in these real alignments were then applied to each of the 540 simulated alignments (see Figure 1), resulting in a set of 5400 EST-like gappy alignments. Because the lengths of the real and simulated alignments differed, the positions and lengths of the gaps in the real alignments were scaled to the length of the simulated alignments (i.e., longer alignments have longer gaps than shorter alignments). Most of the gaps we apply are not from indels but from incomplete sequencing and occur at one end or both ends of a sequence. In real data, such gaps would be expected to increase in size with the length of the unigene, as in our simulation. The ten alignments on which gap patterns were based contained exactly 60 sequences, so one sequence in the gap pattern was used, without replacement, as the template for one sequence in the experimental alignment. For the simulated alignments of ten sequences, only the first ten sequences in the gap patterns were used as templates.

#### Control alignments

For the control alignments, we also used the ten gap patterns described above to delete residues from the 540 full artificial alignments as before, but the residues to be deleted for these controls were either concentrated by column (*column-deleted*), or randomly distributed throughout the alignment (*random-deleted*). For the 5400 column-deleted alignments, columns of characters, i.e., all residues at a given alignment position, were randomly selected without replacement and deleted. In the 5400 random-deleted alignments, the same number of total residues per sequence as in the corresponding gap pattern were independently and randomly selected for deletion, without replacement (Figure 1).

### Masking

The alignment masking algorithm REAP works in two steps by first discarding columns and then discarding sequences. Let *S*_*ijv *_be the score for the aligned residue pair from sequences *i *and *j *in position *v *according to some substitution matrix. In this study, we used the PAM250 substitution matrix [42]. First, REAP evaluates sliding windows of *w *columns along an alignment with *m *columns (residue positions) and *n *sequences. Columns are discarded when the proportion of gap characters exceeds a threshold *g*_*c*_, or when the mean sum-of-pairs score within the window

$$\frac{1}{w}\sum_{v=1}^{w}\sum_{i\neq j}\frac{S_{ijv}}{n(n-1)}$$

is lower than a threshold *s*_*c*_. Counting only those columns that remain, a sequence *i *is discarded when the proportion of gap characters exceeds a threshold *g*_*s*_, or for when the mean sum of pairs score to all other sequences

$$\frac{1}{m}\sum_{v=1}^{w}\sum_{i\neq j}\frac{S_{ijv}}{\left(n-1\right)}$$

is equal to or lower than *s*_*s*_.

To determine the optimal alignment masking parameters, the following REAP parameter values were tested: window sizes of *w *= 3, 6, sum-of-pairs threshold scores of *s*_*c *_= 0.5, 1.0, 1.5, column gap thresholds of *g*_*c *_= 0.1, 0.2, 0.5, and sequence gap thresholds of *g*_*s *_= 0.5, 0.7. Because there is no alignment error in the simulated data, the sum-of-pairs score threshold for eliminating misaligned sequences was not varied but instead held constant at a value of *s*_*s *_= 0.25. All 5400 EST-like gappy alignments were masked with each of the 36 different combinations of variable REAP parameters and the resulting masked alignments were input to phylogenetic analyses as described below.

### Phylogenetic estimation

We computed a phylogeny for each replicate using NJ, MP, and ML. Unrooted NJ phylogenies were computed using the PHYLIP programs Protdist (with the JTT substitution matrix) and Neighbor [43]. ML phylogenies were computed using RAxML-VI [44,45], again with the JTT matrix. All other RAxML settings were defaults. Strict consensus MP phylogenies were computed using TNT [46] with the tree-bisection-reconnection search procedure. Since preliminary tests showed no appreciable difference in the results whether 10 or 500 random addition sequences and 10 or 100 ratchet iterations were used in TNT, we used 10 random addition sequences and 10 ratchet iterations for analysis of the full dataset.

### Statistically correcting for missing data

The SIA algorithm takes an alignment as input. An *overlap graph *is constructed in which each sequence is represented by a node, and the edges between nodes are weighted by the number of shared nongap alignment characters between the two sequences. Using the Bron-Kerbosch algorithm [47], maximal cliques of at least three vertices are identified at a given weight threshold. Cliques represent subsets of vertices in which each pair of vertices is connected by an edge, and these correspond to sets of sequences with sufficient overlap for computation of pairwise distances among all clique members. Each column of the sequence alignment is then assigned to the clique containing the maximal number of sequences with non-gap characters in the column. Columns tied between two or more cliques are assigned to the clique with the fewest total columns (i.e., the blue columns in Figure 4). The columns assigned to a given clique are concatenated to generate a subalignment. Masking is then performed to remove sequences from the subalignment that consist primarily of gaps. For this, we used REAP (described above) with the following parameters: *s*_*c *_= 0.5, *s*_*s *_= 0.25, *g*_*c *_= 0.1, *g*_*s *_= 0.5 and *w *= 3 and discarded subalignments with fewer than three columns. In order to identify cliques with common sequence pairs, a *subalignment graph *is computed in which each subalignment is represented by a node and undirected edges are drawn between subalignments having at least two sequences in common. The connected component that represents the largest number of subalignments is then chosen and the others are discarded. In the absence of any connected components with more than one subalignment, the clique with the largest number of columns is chosen. For subalignments containing more than two columns, pairwise distance matrices are then computed, as above, which results in one or more submatrices.

The submatrices were then combined into one matrix by a linear model in which the distances in each submatrix *k *are scaled by a factor that takes into account (i) the relative rate of substitution for the columns in each submatrix relative to the alignment as a whole and (ii) the relative uncertainty in that estimate as a function of the length of the subalignment. A number of recent papers propose similar methods for combining data from different partitions of a phylogenetic dataset [48,49]. For computational convenience, we computed scaling factors directly from the simulation parameters rather than estimating them from the data. Since the scaling factors are computed without estimation error, the phylogenetic accuracy of this approach will be lower on real data. The details of the model and estimation approach that would be applied to real data are described in a companion publication (F. Cheng, S Hartmann, M. Gupta, J. Ibrahim, T Vision, in prep).

Here, we calculated the relative substitution rate for subalignment *k *based on the average substitution rate of the columns in the subalignment *s*_*k *_and the average substitution rate of all columns in the alignment *s*_*t*_

*r*_*k *_= *s*_*k*_/*s*_*t*_

These rates are known from the ROSE parameters. The scaling factor was then weighted based on the number of columns in a given subalignment

*γ*_*k *_= *r*_*k*_/*w*_*k*_

The weighting term *w*_*k *_is based on that used with real data, and reflects the fact that the precision of the estimate varies with subalignment length. For a subalignment *m*_*k*_, it is computed as

$$w_{k}=\frac{\sqrt{m_{k}}}{\sum_{i=1}^{K}\sqrt{m_{i}}}$$

The motivation for *w*_*k *_is that subalignments with larger number of columns are expected to have less variation in their pairwise distance values.

Pairwise distances that were were not present in any submatrices were imputed using a four-point metric that requires five of the six pairwise distances for four sequences be known and assumes additive tree distances among the sequences [18,39]. The combined matrix with imputed missing values was then used to compute a phylogeny using Neighbor Joining.

### Computing phylogenetic accuracy

We compared the estimated trees with the true trees by measuring the Quartet Distance (*QD*) [31,32] as implemented by Mailund and Christiansen [50]. The *QD *between two phylogenetic trees is the number of quartets, or sets of four sequences, that differ in topology (placement of the internal branch) between them. Since *QD *is dependent on the number of possible quartets, and this differs among comparisons, we calculated standardized quartet distances by dividing *QD *by the total number of possible quartets.

$$stQD=\frac{QD}{\left(\begin{array}{l} n\\ 4 \end{array}\right)}$$

where *n *is the number of sequences in common between the two phylogenies. *stQD *therefore ranges from zero to one.

Different measures for comparing phylogenies exist, but unfortunately all of them can be inadequate in some cases. We chose the quartet distance for this study because it is appropriate when major rearrangements of individual taxa are expected, as was expected for our data [48]. For example, two phylogenies in which only the positions of two taxa are switched (e.g., (A,(B,(C,(D,(E,(F,(G,(H, Z)))))))); (Z,(B,(C,(D,(E,(F,(G,(H, A)))))))); ), have no bipartitions in common, but the corresponding quartet distance still appropriately reflects the similarity of their topologies.

## Authors' contributions

SH and TJV designed the experiments and analyses and wrote the paper. SH performed the computational experiments and analyses. Both authors read and approved the final manuscript.
